# Priming the Semantic Neighbourhood during the Attentional Blink

**DOI:** 10.1371/journal.pone.0012645

**Published:** 2010-09-14

**Authors:** Irina M. Harris, Michael J. J. Little

**Affiliations:** School of Psychology, University of Sydney, Sydney, Australia; Ecole Polytechnique Federale de Lausanne, Switzerland

## Abstract

**Background:**

When two targets are presented in close temporal proximity amongst a rapid serial visual stream of distractors, a period of disrupted attention and attenuated awareness lasting 200–500 ms follows identification of the first target (T1). This phenomenon is known as the “attentional blink” (AB) and is generally attributed to a failure to consolidate information in visual short-term memory due to depleted or disrupted attentional resources. Previous research has shown that items presented during the AB that fail to reach conscious awareness are still processed to relatively high levels, including the level of meaning. For example, missed word stimuli have been shown to prime later targets that are closely associated words. Although these findings have been interpreted as evidence for semantic processing during the AB, closely associated words (e.g., *day-night*) may also rely on specific, well-worn, lexical associative links which enhance attention to the relevant target.

**Methodology/Principal Findings:**

We used a measure of semantic distance to create prime-target pairs that are conceptually close, but have low word associations (e.g., *wagon* and *van*) and investigated priming from a distractor stimulus presented during the AB to a subsequent target (T2). The stimuli were words (concrete nouns) in Experiment 1 and the corresponding pictures of objects in Experiment 2. In both experiments, report of T2 was facilitated when this item was preceded by a semantically-related distractor.

**Conclusions/Significance:**

This study is the first to show conclusively that conceptual information is extracted from distractor stimuli presented during a period of attenuated awareness and that this information spreads to neighbouring concepts within a semantic network.

## Introduction

The purpose of this study was to investigate the relationship between consciousness and depth of processing by examining whether semantic information is extracted during a period of attenuated awareness known as the *attentional blink* [1]. An attentional blink (AB) occurs when two target stimuli are presented within 200–500 ms of each other, under rapid serial visual presentation (RSVP) conditions, and is characterised by an impairment in detecting the second target (T2) following successful identification of the first target (T1). The AB is thought to arise from a combination of depletion of attentional resources and temporal selection processes which prevent T2 from reaching consciousness (see [2] for a review). However, numerous studies have shown that stimuli affected by the AB are nevertheless processed to high levels [3]–[8], consistent with proposals that the AB deficit reflects a relatively late, post-perceptual, stage of processing [9]–[12].

The amount and nature of stimulus processing during the AB can be inferred from priming effects produced by “blinked” stimuli. For example, a number of studies have shown that a missed target can prime a subsequent item that shares the same identity but not necessarily the same perceptual features [8] or an item that is semantically associated with it [4], [5], [6], [7], [8], [13]. Related evidence comes from electrophysiological studies that demonstrated an N400 potential, typically associated with detection of semantic mismatch, in response to missed T2s [4], [7], [14]. Even more remarkably, distractors presented during the AB time window (which one would expect to receive even less attention and conscious processing) have also been shown to prime associated targets [5], indicating that distractors that presumably escape conscious awareness are also processed to the level of meaning. Maki and colleagues [5] found that a priming distractor (PD) presented just before T2 produced significant associative priming, although this priming was short lived and disappeared if more distractors intervened between the PD and T2 (i.e., it seemed to last up to 200 ms).

Although the evidence reviewed above has been ascribed to semantic processing of stimuli outside awareness, there is some fluidity in the definition of semantic information or semantic relationships between stimuli. For example, some refer to the relationship between equivalent words in different languages as semantic [15]. Others use category sets to cue the targets (e.g., [16]), and at least one paper has referred to the relationship between a letter presented in upper case and the same letter presented in lower case as a semantic relationship [17]. But by far the most common manipulation employed in studies of semantic priming within the AB has been the use of word associations (e.g., *doctor-nurse*, *day-night*, *foot-shoe*, etc). This limits the conclusions drawn from these studies about semantic activation in the absence of awareness, because word associations (and other relationships such as translation priming), while possibly reflecting true conceptual information, might also rely on specific, well-worn, lexical associative links which enhance attention to subsequent items. If we are to conclude that true semantic information is accessed outside of awareness, then, ideally, we would like to see priming based on categorical relationships, where those categories are not small and well rehearsed or linked by associative relationships. Furthermore, true semantic information is independent of stimulus domain and stimulus format, so we would expect to see this kind of priming for both word and picture stimuli.

With this in mind, the present experiments employed prime-target stimulus pairs that had a high degree of semantic overlap combined with low associability of their word forms. We used both word (Experiment 1) and corresponding picture stimuli (Experiment 2) and measured priming from a distractor presented just before T2, either in the depth of the blink (the second stimulus after T1) or outside the temporal window of the AB (the 6th stimulus after T1).

### Defining semantic similarity

**Figure 1:**
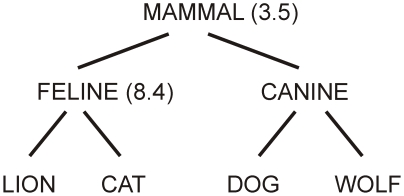
A schematic of a lexical tree showing edge-based and node-based approaches to measuring the semantic distance between *cat* and *lion* versus *cat* and *dog*. The edge-based approach counts along the shortest path between two words, while the node-based approach relies on the information content of their nearest common superordinate. By both these measures, *cat* is closer to *lion* (2 edges and a shared information content of 8.4) than it is to *dog* (4 edges and a shared information content of 3.5). The values given here are merely for illustrative purposes and do not represent true information measures.

For the purposes of this study, semantic similarity was defined in terms of underlying semantic concepts, independent of factors such as co-occurrence in bodies of text or free association by subjects. There have been several approaches to the problem of assessing semantic distance based on electronic taxonomies of words and concepts [18]. Node-based approaches use the information content of individual concept nodes, based on their frequency in a corpus of text. In these approaches the distance between two concepts is determined by the information content of their lowest common superordinate, which is inversely related to probability of occurrence in a body of text. So the more common a concept is, the lower its information content [18], [19]. For example, as illustrated in Figure 1, the information content of the concept “feline” might be 8.4, whereas the information content of “mammal” might be 3.5 – that is, the concept “mammal” occurs more frequently and, thus, carries less information. According to the node-based approach, *cat* is more similar to *lion* than to *dog*, because the superordinate node “feline” shared by *lion* and *cat* has higher information content than the nearest superordinate node (“mammal”) shared by *cat* and *dog*. The node based approach is conceptually simple and its results produce a correlation of approximately 0.79 with human ratings of the same words and concepts. In contrast, edge-based approaches examine the distance between nodes in terms of the number of edges one has to travel to get from one to the other (see Figure 1). This approach makes intuitive sense, since it reflects the hierarchy and structure of our conceptual taxonomy, but requires careful adjustment of the weights of the edges between nodes to account for heterogeneity within the conceptual hierarchy. Concepts at the top of the hierarchy are likely to be more coarsely defined than concepts lower down, and a naïve edge count will therefore tend to underestimate distance at the top or overestimate distance at the bottom of the hierarchy. Here, we adopted Jiang and Conrath's [18] combined approach which uses elements of both.

Jiang and Conrath's (JCN) semantic distance algorithm uses information content as a weighting factor for edges. To determine these weightings, Jiang and Conrath examined the conditional probabilities of the appearance of particular concepts in text corpora given the appearance of their superordinates (e.g., the probability that a particular word refers to the concept “dog” given that it is known to refer to the concept “canine”). Some words are very common exemplars of their superordinate categories, and others less so (e.g., “dog” vs “wolf”). The JCN algorithm incorporates this understanding to weight the links between these concepts accordingly. Comparison of the JCN algorithm with human judgments shows that it strongly reflects human impressions of semantic distance, more so than either the edge-based or node-based approaches alone. The JCN algorithm achieved an optimal correlation with human judgments of 0.87, compared to a human replication correlation of 0.88 and correlations of 0.82 and 0.60 for the node-based and edge-based approaches respectively [18].

Maki et al. [19] used the JCN algorithm to compute semantic distances for a set of 49,559 pairs of nouns and verbs and combined these in a database with other common word norms for the same pairs, including forward and backward associative strength. This database provided us with the opportunity to identify word pairs that have relatively close semantic relationships uncontaminated by significant associative links.

## Methods

### Experiment 1

The purpose of Experiment 1 was to find out whether or not word distractors presented in the AB (at Lag 2 or 213 ms after T1) can prime T2 on a purely semantic basis, according to the JCN measure of semantic distance. The priming effect obtained from blinked distractors was compared to that obtained from a distractor presented at a temporal lag long enough to avoid the effects of AB (Lag 6 or 636 ms after T1).

#### Participants

Twenty-five undergraduate students at the University of Sydney (8 women and 17 men, aged 17–41) participated in exchange for course credit. All subjects gave written informed consent for participation, and all reported being native English speakers and having normal or corrected-to-normal vision. The procedures were approved by the Human Research Ethics Committee of the University of Sydney.

#### Materials and Apparatus

The T1, T2 and priming distractor (PD) words for this experiment were concrete nouns drawn from the set of semantic distance norms produced by Maki et al. [19] based on the JCN algorithm. The PD and T2 items were selected in pairs based on their JCN distances. The closest pair had a JCN distance of 0.54 (“van” and “wagon”) and the furthest pair had a distance of approximately 6.61 (“truck” and “caravan”); these distances are both considered close. The pairs were selected without regard to semantic domain, and included a mix of living and non-living items. Maki et al. also provided associative norms for each word pair in their database, drawn from Nelson, McEvoy and Schreiber [20]. The average forward associative strength from prime to target for the words used in this experiment was 0.25%, with individual values ranging from 0% to 4.7%. The forward associative strength from word A to word B is defined as the percentage of participants who said word B in response to word A in the normative study conducted by Nelson and colleagues; 0 indicates that no participants produced word B in response to word A, i.e. that there is no evidence of an association between these words. Thus the priming word pairs were semantically close without being closely associatively related. T1 words were initially selected for their high semantic distances from particular T2 items that had already been included in the stimulus set. The pairing of T1 and T2 items in each version of the experiment was subsequently randomised to prevent repetition of target pairs. The general distractor set consisted of 72 abstract nouns generated by the experimenter, with no systematic semantic relationship with the target words. Stimuli for the practice items consisted of personal names.

All stimuli were presented using DMDX presentation software [21] on a 19” monitor with a refresh rate of 11.764 ms per frame (85 Hz), on a white background. The words appeared on screen with a height of approximately 8 mm and a length of approximately 25 mm. Participants viewed the stimuli from a distance of around 60 cm, and the stimuli therefore subtended approximately 2.4° of visual angle.

#### Design

**Figure 2:**
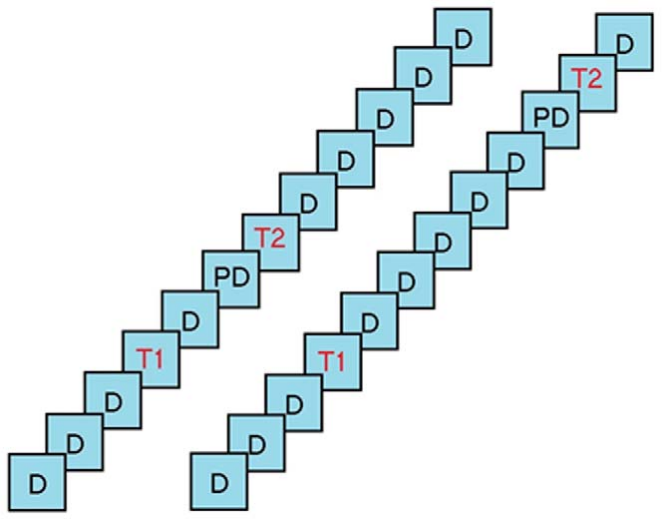
Trial structure in Experiments 1 and 2. Rapid Serial Visual Presentation sequences contained two targets (T1 and T2) and 12 distractors. T2 followed T1 with a lag of 3 items (left) or 7 items (right). The priming distractor (PD), when present, occurred just before T2.

There were four conditions defined by two variables: PD-T2 relatedness and serial lag between T1 and T2. Each trial consisted of twelve distractor words presented in black and two target words presented in red. On half the trials, T2 was preceded by a specially selected PD that was semantically related to it (related conditions) and on the other half by a randomly selected member of the general distractor pool (unrelated conditions). T2 appeared at Lag 3 on half the trials and at Lag 7 on the other half, relative to T1, and the PD was always the item preceding T2 (see Figure 2).

Each of the 36 T2 items appeared once in each of the four conditions (related Lag 3, unrelated Lag 3, related Lag 7 and unrelated Lag 7), giving a total of 144 experimental trials for each subject. To reduce the likelihood of order effects, four different versions of the experiment were created using multiple randomisations of T1-T2 pairing and non-priming distractor distribution.

#### Procedure

Participants were seated comfortably in front of a computer monitor at a distance of approximately 60 cm. They were instructed to report the identities of the two red target words in each RSVP stream. Participants completed five practice trials consisting of personal name targets and distractors, followed by the 144 experimental trials. Each trial began with a display of 10 hash symbols appearing in the centre of the screen for 588 ms, which was then replaced by the RSVP stream. Each RSVP sequence consisted of 14 items: 3 general distractors, T1, 1–5 general distractors, the PD, T2 and finally 3–7 general distractors to finish the sequence. Each stimulus appeared for 106 ms.

The experimenter recorded the participants' verbal responses. No feedback was given and participants pressed the spacebar at their own pace to proceed to the next trial. The dependent variable of interest was accuracy of reporting T2, conditional on correct report of T1 (T2|T1), which is the standard way of measuring AB.

### Experiment 2

If the results of Experiment 1 are based on genuinely semantic relationships then the spreading activation giving rise to priming in that experiment should be occurring in semantic networks that are not confined to particular stimulus formats or modalities. This hypothesis was tested in Experiment 2 using objects stimuli, instead of words.

#### Participants

Twenty-five undergraduate and postgraduate students from the University of Sydney (13 women and 12 men, aged 18–27) participated either for course credit or $10 payment, or as unpaid volunteers. All subjects gave written informed consent for participation, and all reported being native English speakers and having normal or corrected-to-normal vision. None had participated in Experiment 1. The procedures were approved by the Human Research Ethics Committee of the University of Sydney.

#### Materials

The stimuli for this experiment were sets of images chosen to represent the same concepts as the words in Experiment 1. Due to difficulties in finding images corresponding to some of the words, the original set of 36 T1s, 36 T2s and 36 PDs was reduced to 28 of each stimulus type. The PD-T2 pairs in this reduced stimulus set had JCN distances ranging from 0.54 to 6.61, with a mean of 2.55. Forward associative strength between PDs and T2s ranged from 0 to 4.7% with a mean of 2.3%.

The target and PD stimuli consisted of colour images of objects whose names were used as targets or PDs in Experiment 1. The image set was obtained partly from the Photo-Object database (Hemera Inc, Canada) and partly from Google Images. Each object was presented isolated on a uniform mid-grey background. All of the resulting images were resized to 300 pixels in their longest dimension. The final images measured up to 90 mm×90 mm. T1 and T2 images were kept in colour, while PD images were converted to greyscale. Thirty-six different masks were created, consisting of 300×300 pixel images composed of scrambled elements from the 84 target and PD images. The masks were then partially desaturated, making each one a mixture of coloured and greyscale patches (see Figure 4 for examples of stimuli). The stimuli for the practice items at the start of the experiment consisted of the same masks and six images of construction tools which were not used as part of the main experiment.

#### Design

Two independent variables were crossed in a 2×3 within-subjects design. T1-T2 lag had two levels (Lag 3 and Lag 7) and PD-T2 relatedness had three levels (PD andT2 were related objects, PD and T2 were unrelated objects, or PD was a mask stimulus). Participants completed 168 experimental trials, divided into the six conditions. All participants completed all conditions, and each participant saw each of the 28 T2 items once in each condition. In order to prevent short-term association from influencing the results, the same unrelated PD-T2 pairs were used in both the short and long lag conditions of any given version of the experiment. Thus each PD-T2 pairing, whether related or unrelated, appeared twice for each participant. The pool of masking images was randomised so that no mask was repeated on a given trial.

#### Procedure

The procedure was identical to that of Experiment 1 except as noted here. The practice run consisted of only three trials and participants were instructed that the target items were distinct from the rest of the stream because they were fully coloured, whole objects. There were 168 experimental trials, and each stimulus appeared for 71 ms (this reduction in exposure duration relative to Experiment 1 was necessary to avoid ceiling effects and obtain a level of accuracy comparable to Experiment 1).

## Results

### Experiment 1

Experiment 1 investigated whether or not word distractors presented in the AB (at Lag 2 or 213 ms after T1) can prime T2 on a purely semantic basis. The priming effect obtained from blinked distractors was compared to that obtained from a distractor presented at a temporal lag long enough to avoid the effects of AB.

Five participants were excluded because they did not show an attentional blink, given that in this study we are specifically interested in the amount of processing undergone by stimuli presented during the AB. The size of AB was calculated by subtracting T2|T1 accuracy in the unrelated Lag 3 condition from T2|T1 accuracy in the unrelated Lag 7 condition and any participant with an AB size <10% was excluded.

**Figure 3:**
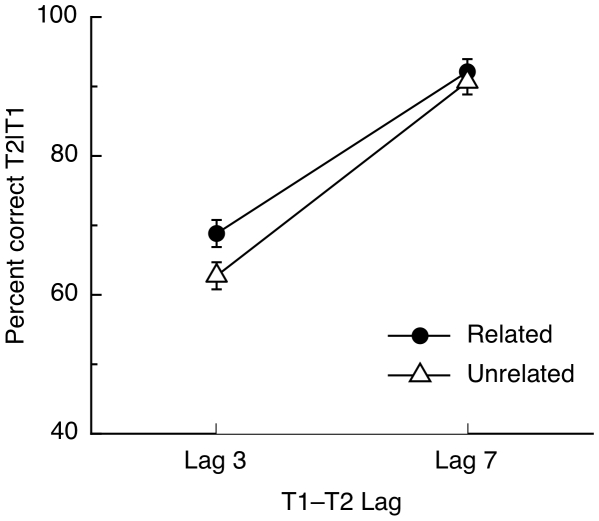
Results of Experiment 1. Mean T2|T1 accuracy for the related and unrelated priming conditions, plotted as a function of the lag separating the two targets. Error bars represent standard error of the mean difference (within-subjects) between the related and unrelated trials at each lag.

Figure 3 shows mean T2|T1 accuracy plotted as a function of condition. A 2×2 repeated-measures ANOVA showed a significant effect of prime-target relatedness, *F*(1,19) = 5.91, *p* = 0.025, η_p_^2^ = .237, a strong effect of lag indicating an attentional blink, *F*(1,19) = 63.70, *p*<0.001, η_p_^2^ = .770, and a significant interaction between lag and prime-target relatedness, *F*(1,19) = 4.61, *p* = .045, η_p_^2^ = .195. Pairwise comparisons between the related and unrelated conditions confirmed a significant priming effect at Lag 3, *t*(19) = 3.12, *p* = .006, but no priming at Lag 7, *t*(19)<1.

**Figure 4:**
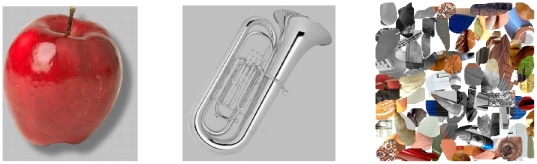
Sample images from the stimulus sets for Experiment 2. A target (left, presented in full color), a distractor (centre, presented in greyscale) and a scrambled image mask (right).

These results demonstrate that distractor words presented in the depth of the blink can produce semantic priming when the primes and targets have close JCN semantic distance, but low associative strength, implying that stimuli presented during the AB activate semantic networks and this activation spreads to neighbouring concepts within the network. The lack of priming in the Lag 7 condition is possibly due to a ceiling effect, given that performance at this lag was quite high even in the unrelated trials. An alternative explanation is that distractors presented outside the AB underwent a certain amount of suppression and this reduced any priming that would have otherwise been present. A number of studies have shown that distractors in the RSVP stream are inhibited in order to facilitate target selection [3], [22]–[24], but this is only apparent when attention is available (as is the case at long lags) but not during the AB [3], [25].

### Experiment 2

Experiment 2 tested whether the priming seen in Experiment 1 from a distractor presented during the AB could be obtained using object stimuli instead of words.

**Figure 5:**
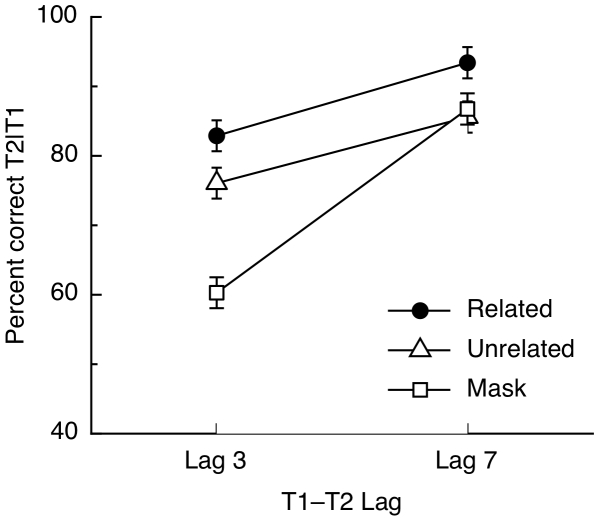
Results of Experiment 2. Mean T2|T1 accuracy for the related, unrelated and mask (baseline) priming conditions, plotted as a function of the lag separating the two targets. Error bars represent the within-subject s.e.m for the main effect of priming [33].

As in Experiment 1, participants with an AB magnitude <10% were excluded from the analysis. This resulted in the exclusion of three participants, leaving 22 for further analyses. Figure 5 shows mean T2|T1 accuracy data for this experiment, plotted as a function of T1-T2 lag and priming condition. A 2×3 repeated-measures ANOVA showed a significant effect of priming condition, *F*(2,42) = 28.55, *p*<.001, η_p_^2^ = .576. Pairwise comparisons of conditions confirmed significantly higher accuracy in the unrelated than in the mask condition (*p* = .014, Bonferroni-corrected; mean accuracy for the unrelated condition  = 81%, mean accuracy for the mask condition  = 74%) and higher accuracy in the related than in the unrelated conditions (*p*<.001, Bonferroni-corrected; mean accuracy for related condition  = 88%). There was also a significant effect of lag, *F*(1,21) = 49.43, *p*<.001, η_p_^2^ = .702, consistent with an AB, as well as a significant interaction between priming condition and lag, *F*(2,42) = 23.48, *p*<.001, η_p_^2^ = .528. Paired t-tests were carried out to break down the interaction and demonstrated significantly better performance in the related than in the unrelated condition for both lags (*t*s>2.94, *p*s<.008) and significantly better performance in the unrelated compared to the mask condition at Lag 3, *t*(21) = 5.68, *p*<.001, but no difference between the unrelated and mask conditions at Lag 7 (*t*<1). Accuracy was also significantly higher in the related condition than in the mask condition for both lags (*t*s>3.41, *p*s<.003).

The results of this experiment replicate those of Experiment 1 using pictures of objects instead of word stimuli. We again found priming from a semantically-related distractor object presented during the AB, but unlike in Experiment 1, we also found semantic priming in the long lag condition. In addition, in the present experiment we observed a facilitation in T2 report when an unrelated object was presented immediately before it, compared to when the preceding item was a pattern mask similar to the other distractors in the RSVP stream. The latter finding is consistent with some previous reports that showed a short lasting cueing effect when a target is preceded by a stimulus from the same general category [26] or a stimulus that shares target features [27]. This cueing benefit is typically only manifest during a period of attentional blink, when attentional engagement may be affected, and not outside the blink when attentional processes operate normally [27]. Crucially, the semantic priming that is of primary interest here was over and above any such cueing effects.

As discussed previously, the absence of semantic priming at the long T1-T2 lag in Experiment 1 may be due to a ceiling effect, as accuracy at Lag 7 was very high in that experiment, whereas in Experiment 2 performance at Lag 7 was lower, providing some headroom for priming to be observed. This may explain why we saw semantic priming at the long lag here. Another possible explanation is that the difference between the two experiments is due to stronger semantic spreading activation for pictures compared to words. Alternatively – though these two explanations are not mutually exclusive – there was less distractor suppression at the longer lag in this experiment because the shorter stimulus durations (71 ms in Experiment 2, compared to 106 ms in Experiment 1) meant that a distractor occurring at Lag 6 was only 426 ms after T1 and, thus, still partially affected by the AB. This may have resulted in less effective suppression and, consequently, a larger priming effect (see [3] for related findings).

Regardless of the reason for priming at the long lags, the critical finding of this experiment is that semantic priming, as measured by semantic distance between prime and target concepts, was obtained using object stimuli presented in the depth of the AB.

## Discussion

This study is the first to demonstrate semantic priming based on a pure measure of semantic distance (as opposed to word associations) from a distractor stimulus affected by the attentional blink. In two experiments, we showed that such priming can be obtained for both word and picture stimuli, emphasising that the priming effect is conceptual in nature and not tied to processes specific to a particular stimulus format.

All studies of semantic priming in the AB to date have used word stimuli and defined the relationship between the prime and target stimuli in terms of strength of word associations. This approach is therefore contaminated by well-worn links between lexical representations and might not represent true conceptual associations. In contrast, the approach we employed here emphasised categorical relationships between stimuli, while controlling for word associations. The results provide clear evidence that categorical information is extracted from ignored stimuli (distractors) and spreads to neighbouring concepts within the semantic network. This spreading activation occurs despite the attenuated attention and conscious awareness induced by the AB. On the basis of the results of Experiment 2 alone one might be tempted to attribute the priming effect seen here to visual features shared by items with a close semantic distance (e.g., wheels for “wagon” and “van”; legs and snout for “dog” and “wolf”), rather than to the activation of individual concepts, which some researchers have argued cannot occur without attention [28]. However, this argument does not hold for the priming obtained with the word stimuli used in Experiment 1. The only way to explain the semantic priming obtained for words is by specific identification of individual concepts in the semantic network and spreading activation from this to another concept. That is not to say, however, that concepts are represented in a localist fashion in the brain, as individual items sitting on the branches of taxonomic trees [29], as they are often depicted in diagrams of semantic hierarchies and neighbourhoods (e.g., see Figure 1). A semantic neighbourhood may, instead, consist of a pool of perceptual and conceptual features that are shared to some extent by different individual concepts, with closer neighbours sharing a greater number of these features [30], [31]. Thus, when conceptual information pertaining to one item is activated, this information spreads rapidly, and seemingly in the absence of attention and awareness, to its closest neighbours in semantic space.

### Implications for models of AB

The present results are consistent with models of RSVP processing that advocate automatic processing of conceptual information for all stimuli, be they targets or distractors, with a subsequent capacity-limited stage in which targets are consolidated in visual short-term memory prior to report [9]. Other models of the AB which view the phenomenon as being due to inhibitory mechanisms triggered by the identification of the first target or the distractor immediately following the first target [1], [11] have difficulty accounting for the present results. According to such models, when T1 is identified this causes an attentional gate [1], or “bouncing” mechanism [11], to block any subsequent items (with the sole exception of the item immediately following T1, the so-called *lag 1 sparing* effect) from further processing. The present results speak against such an inhibitory mechanism operating during the period of the AB, given the clear priming obtained from a distractor item presented at lag 2 (see also [3], [5], [32]). However, they support Dux and Harris' [3], [25] hypothesis that distractors are not effectively inhibited during the attentional blink, allowing their representations to influence the processing of subsequent items at a number of different levels of processing, ranging from shared perceptual features to high-level conceptual information.
